# Surgical management of duodenal perforation complicated with retroperitoneal abscesses and paralytic ileus: a case report

**DOI:** 10.3389/fsurg.2026.1745803

**Published:** 2026-07-21

**Authors:** Qingtao Zhao, Zhenguang Bian, Jingzhou Wang, Jian Chen

**Affiliations:** 1 Department of General Surgery, Bethune International Peace Hospital, Shijiazhuang, Hebei, China; 2 Department of Gastroenterology, Bethune International Peace Hospital, Shijiazhuang, Hebei, China

**Keywords:** case report, duodenal perforation, paralytic ileus, retroperitoneal abscesses, surgical management

## Abstract

Duodenal perforation is a critical gastrointestinal emergency that poses significant morbidity and mortality risks globally. This case report examines a unique presentation of duodenal perforation in a 78-year-old male patient who initially exhibited chronic abdominal pain and paralytic ileus, a manifestation not commonly associated with this condition. The patient underwent coronary stent implantation 5 years ago following myocardial infarction (MI) and has been on long-term oral aspirin therapy since. Upon admission, he displayed worsening symptoms, which included distension, inability to eat, and minimal bowel movement. Initial management strategies involving fasting, acid suppression, and antibiotics failed to ameliorate his condition. A follow-up abdominal CT scan revealed paralytic ileus, duodenal edema, and ultimately, retroperitoneal gas and abscess. An urgent laparoscopic exploration confirmed duodenal perforation, leading to surgical intervention that included suturing the perforation and draining the retroperitoneal space. This case underscores the importance of recognizing atypical presentations of duodenal perforation, advocating for heightened awareness and prompt intervention to mitigate serious complications associated with delayed diagnosis.

## Introduction

Duodenal perforation is a serious gastrointestinal emergency with an incidence that varies but remains a notable cause of morbidity and mortality worldwide (1). Specific segmental duodenal perforation can lead to retroperitoneal infection. Retroperitoneal infection is a persistent and widespread infectious disease that is difficult to treat (2). It is usually caused by secondary complications such as inflammation, damage, or perforation of adjacent organs in the retroperitoneal space.

Cases where duodenal perforation manifests primarily as chronic abdominal pain with complications such as paralytic ileus have not been reported. This atypical presentation can lead to delays in diagnosis and treatment, thereby increasing the risk of adverse outcomes. In this case report, we present a unique instance of a duodenal perforation that initially presented with paralytic ileus and was associated with a retroperitoneal abscess. This case highlights the importance of recognizing atypical presentations of duodenal perforation, especially in patients with chronic gastrointestinal symptoms. Through this report, we aim to increase awareness of the diverse clinical manifestations of duodenal perforation and underscore the need for a high index of suspicion in such cases. Additionally, we discuss the management strategies employed and the patient's subsequent recovery, contributing to the literature on this critical condition.

## Case report

A 78-year-old male presented with a history of recurrent upper abdominal pain, which had been described as cyclic, occurring predominantly during the night while fasting or after meals, and he had been on long-term omeprazole therapy. The patient underwent coronary stent implantation 5 years ago following myocardial infarction (MI) and has been on long-term oral aspirin therapy since. Additionally, the patient has chronic comorbidities such as hypertension and diabetes mellitus but no history of abdominal surgery. Five days prior to admission, the patient experienced abdominal pain, distension, nausea, vomiting and absence of fever. He underwent an upright abdominal x-ray at an outpatient clinic, which indicated signs of paralytic ileus. CT scan showed partial dilation of the small intestine, ruling out cholecystitis, pancreatitis, appendicitis, and intestinal tumors. Initial management included gastric acid suppression, antibiotics, and parenteral nutrition; however, the patient's symptoms had not yet been relieved.

**Figure 1:**
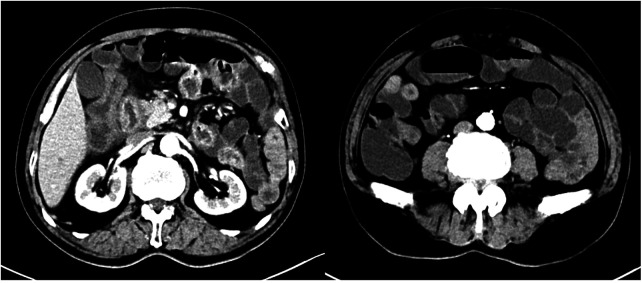
Abdominal CT scan showing paralytic ileus and duodenal edema.

**Figure 2:**
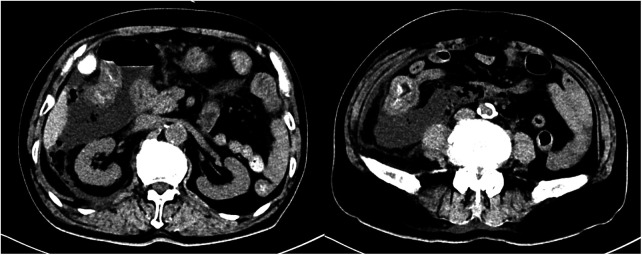
Abdominal CT scan showing retroperitoneal gas and fluid accumulation.

Upon admission, the patient's nausea and vomiting had alleviated, but abdominal distension and difficulty in eating persisted. Physical examination revealed diffuse abdominal tenderness without significant guarding or rigidity. Bowel sounds were hypoactive. Murphy's sign was negative. Laboratory investigations revealed a white blood cell count of 15.5 × 10⁹/L (Ref. range: 3.5–9.5 × 10⁹/L), a procalcitonin level of 0.04 ng/mL (Ref. range: 0.02–0.5 ng/mL), and normal serum and urine amylase levels. We repeated an abdominal CT and ultrasound scan within 24 h of admission, which confirmed the presence of paralytic ileus along with duodenal edema (Figure 1). The possibility of acute pancreatitis, cholecystitis, and ischemic bowel disease was also ruled out. In the absence of surgical indications, we continued to administer proton pump inhibitors(PPIs), escalate antibiotic therapy, and provide parenteral nutrition. On the third day, the patient's condition worsened despite ongoing management, with the development of severe abdominal pain, fever, tachycardia, and hypotension. A repeated abdominal CT demonstrated gas within the abdominal cavity and retroperitoneal fluid accumulation (Figure 2).

**Figure 3:**
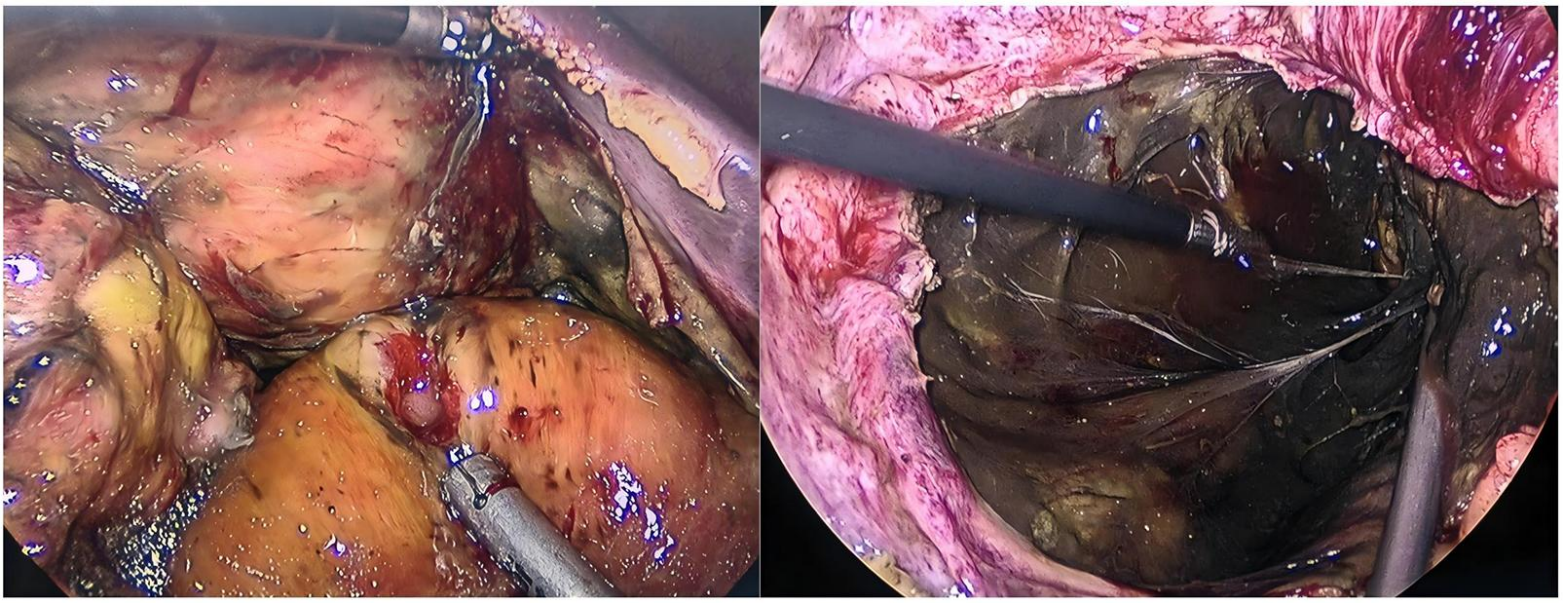
Intraoperative findings revealed a duodenal perforation and retroperitoneal abscesses.

An urgent laparoscopic exploration was performed, which revealed significant adhesion of the omentum, colon transversum and duodenum. Kocher's operation was performed to fully expose the second segment of the duodenum and the pancreatic head area. There was a 0.8-cm perforation on the lateral wall of the second segment of the duodenum, that was draining significant ilious output (Figure 3). Edema of the tissue surrounding the perforation was observed in the absence of mucosal rigidity, consistent with the characteristic presentation of acute perforation complicating chronic duodenal ulcers. The duodenal perforation was repaired with absorbable sutures and reinforced with an omental patch secured to the seromuscular layer. Subsequently, incision was made posterior to the ileocecal region to access the retrocolic space，which uncovered the abscess that extended from the posterior ileocecal region to the duodenum. After thorough debridement of retroperitoneal abscess and infected tissues, two drains were placed in the retrocolic space and site of perforation to facilitate effective drainage.The patient tolerated the procedure well, had an uneventful hospital stay, and successfully advanced their diet. Regarding measures to prevent recurrence, we advised the patient to take oral PPIs after discharge and to regularly follow up for Helicobacter pylori and gastroscopy.

Preoperative gastroscopy was contraindicated due to paralytic ileus. Given the emergency nature of the surgery and the patient's stable postoperative recovery, no tests for Helicobacter pylori (such as C-14 urea breath test or gastric mucosal biopsy) were performed during hospitalization. However, at the one-month postoperative follow-up, C-14 urea breath test yielded negative results. The follow-up gastroscopy results showed chronic duodenal ulcer in the healing stage, ruling out the possibility of tumor. This also confirmed our intraoperative description of the intestinal wall at the perforation site. This case underscores the atypical presentation of duodenal perforation manifesting primarily as paralytic ileus, emphasizing the need for timely diagnosis and intervention to prevent serious complications.

## Discussion

Approximately 10% of patients presenting with upper abdominal pain in a primary care setting have a peptic ulcer as the cause of their symptoms. Complications of peptic ulcer include bleeding (73% of patients) and perforation (9% of patients) (3). There has been a relative dearth of studies on retroperitoneal abscesses caused by duodenal perforation in the past decade. A comprehensive review revealed that only 17 instances of retroperitoneal abscesses secondary to duodenal ulcer perforation were documented across ten articles (4–13) published between 1966 and 2024.In most cases, patients present with typical symptoms of abdominal pain, fever, acute peritonitis, and imaging findings of retroperitoneal fluid accumulation and pneumoperitoneum. However, atypical presentations, such as the paralytic ileus observed in our case, are exceedingly rare and pose significant diagnostic challenges. The literature describes several non-classical manifestations. For instance, perforation can mimic appendicitis, as in Valentino syndrome, where leaked contents track to the right lower quadrant (14). In pediatric populations, cases have been reported where perforation presented as an acute abdomen without typical radiographic signs of pneumoperitoneum (15, 16). Furthermore, perforations may be indolent, presenting initially as an anterior abdominal abscess (17) or, as in cases of retroperitoneal perforation, with subtle symptoms leading to delayed diagnosis and abscess formation (13, 18). Our case is distinct from these reports, as the patient's primary and persistent manifestation was paralytic ileus without any localizing signs of peritonitis or a palpable mass, a presentation not prominently featured in the reviewed literature.

The unique clinical picture in our patient can be attributed to the specific location and nature of the perforation. The 0.8-cm perforation was located on the lateral wall of the second part of the duodenum, with the leak contained within the retroperitoneal space. This likely resulted in chemical irritation of the retroperitoneal nerves and the celiac plexus, leading to reflex intestinal paralysis rather than the typical generalized peritonitis seen with anterior wall perforations. This mechanism is supported by reports of retroperitoneal duodenal perforations, which often present with non-specific symptoms and are frequently diagnosed late (19, 20). The patient's long-term use of aspirin, a non-steroidal anti-inflammatory drug (NSAID), and proton pump inhibitors may have further masked symptoms by suppressing inflammation and ulcer pain. This case underscores the critical need for clinicians to consider an atypical duodenal perforation in the differential diagnosis of unexplained paralytic ileus, particularly in elderly patients with a history of NSAID use, and highlights the importance of timely repeat imaging and a low threshold for surgical exploration.

This case highlights a critical diagnostic pitfall: the failure to recognize an atypical duodenal perforation when it presents as paralytic ileus. The initial CT finding of “duodenal edema” was erroneously interpreted as a non-specific inflammatory change rather than a potential harbinger of a sealed-off perforation. This diagnostic error is common, as radiologists often focus on the more obvious finding of bowel dilation while overlooking subtle signs of retroperitoneal pathology (21). The case emphasizes that for elderly patients on long-term NSAIDs like aspirin, the combination of paralytic ileus and even minor duodenal wall changes on CT should trigger a high index of suspicion for a contained retroperitoneal perforation. The CT features of perforation include discontinuity of the duodenal wall and the presence of extraluminal air or extravasated oral contrast. However, extraluminal air does not always appear in all imaging features. One case report described the imaging morphology of a lesion located in the retroperitoneum (12). The decision to operate was ultimately dictated by the patient's clinical deterioration and the appearance of pneumoperitoneum, underscoring that a “wait-and-see” approach is dangerous in this context; a low threshold for repeat imaging and early surgical consultation is paramount.

Strategies involving radiographically guided percutaneous drainage, rational use of antibiotic agents, and nutritional interventions are the primary approaches used to treat retroperitoneal abscesses (22). The management of duodenal perforations with retroperitoneal abscesses should focus on both perforation repair and thorough exploration of the retroperitoneal space. We emphasize the necessity for adequate debridement and drainage of infected areas to prevent complications and improve outcomes. Although effective management of the retroperitoneal abscess is important, treating the primary etiology remains equally critical. The selection of a surgical strategy depends largely on the specific site and dimensions of the perforation. In cases where the defect is situated near the gastric pylorus, performing a truncal vagotomy combined with pyloroplasty represents a viable therapeutic option. However, for the majority of small perforations occurring elsewhere within the duodenum, closure utilizing a Graham patch is the standard approach. Based on our experience, for smaller duodenal perforations, direct full-thickness suture followed by suturing the omentum to the seromuscular layer for reinforcement can also achieve good results.

From a pathophysiological perspective, this case supports a novel mechanism for the development of paralytic ileus secondary to duodenal perforation. We propose that the tiny, 0.8-cm perforation on the lateral duodenal wall allowed a slow, continuous leak of duodenal contents into the retroperitoneal space. This chemical insult, rather than causing a diffuse bacterial peritonitis, specifically stimulated the celiac plexus and retroperitoneal sympathetic nerves, leading to a reflex inhibition of intestinal peristalsis. This neurogenic ileus explains the preserved bowel sounds early in the course, as the paralysis was functional rather than due to mechanical obstruction. Additionally, infections occurring in the retroperitoneal area may result in paralytic ileus, primarily via processes such as inflammation and neural pathways. These mechanisms can adversely affect both the vascular supply and neuronal signaling (23). This case provides a clinical correlate to this theoretical pathway and adds a critical nuance to the understanding of how a contained, retroperitoneal duodenal leak can manifest systemically through neural pathways, a concept that warrants further investigation in future studies.

This case serves as a critical reminder that atypical duodenal perforation must be considered in the differential diagnosis of unexplained paralytic ileus, particularly in elderly patients with a history of long-term aspirin use. The absence of classic peritoneal signs, coupled with the misleading initial CT finding of duodenal edema, created a diagnostic trap that nearly resulted in a fatal delay. The successful outcome in our patient hinged on a high index of suspicion, prompt repeat imaging upon clinical deterioration, and decisive surgical intervention. This case reinforces the principle that in this specific patient population, a “wait-and-see” approach is hazardous; a low threshold for repeat CT imaging and early surgical consultation is paramount to avoid catastrophic outcomes.

Several limitations of this report warrant acknowledgment. As a single case study, the findings cannot be generalized to all populations. Preoperative Helicobacter pylori testing was not feasible due to the patient's ileus, and long-term follow-up data on ulcer recurrence are limited. Furthermore, the proposed neurogenic mechanism for ileus, while plausible, remains speculative and requires validation through experimental models or larger clinical series. Despite these constraints, this case offers valuable clinical insight into a rare presentation of a common surgical emergency. It underscores the importance of maintaining diagnostic vigilance for contained retroperitoneal duodenal perforations in at-risk patients and highlights the need for future studies to elucidate the pathophysiological link between retroperitoneal duodenal leaks and reflex paralytic ileus.

## Data Availability

The original contributions presented in the study are included in the article/Supplementary Material, further inquiries can be directed to the corresponding author.
