# Supplementary material for: Surgical management of duodenal perforation complicated with retroperitoneal abscesses and paralytic ileus: a case report
Source: Front Surg. 2026 Jul 21;13:1745803. doi: 10.3389/fsurg.2026.1745803 (PMC13433849; doi:10.3389/fsurg.2026.1745803)
Supplement: Supplementary file 1 [file Table1.docx]

| **Time** | **Medical Incident** | **Key Clinical Data** | **Imaging/Laboratory Results** | **Intervention** | **Antibiotic regimen** |
| --- | --- | --- | --- | --- | --- |
| Day 1 | Pre-admission treatment | abdominal distension,nausea,poor appetite,fatigue.  T:36.5℃;HR:87bpm;BP:134/83mmHg | WBC:7.5×10⁹/L; CRP:48mg/L;  X-ray:paralytic ileus without gas in the abdominal cavity.  CT: Partial small bowel dilation, ruling out cholecystitis, pancreatitis, appendicitis, and intestinal tumors. | PPIs,cephalosporin antibiotics,rehydration |  |
| Day 5  09:30 | Emergency admission | Epigastric pain,abdominal distension,nausea,vomiting,poor appetite,fatigue. T:37.2℃;HR:96bpm;BP:121/76mmHg | WBC:15.5×10⁹/L; CRP:121mg/L;  Lactate:1.4mmol/L;Procalcitonin:0.04ng/mL | Fasting,rehydration,PPIs,cephalosporin antibiotics | Cefotaxime 1g q8h |
| Day 5  14:00 | Enhanced CT scan | Epigastric pain,Nausea,and vomiting had alleviated. | CT:paralytic ileus, duodenal edema | Parenteral nutrition | Cefotaxime 1g q8h |
| Day 6  08:30 | Conservative treatment | Abdominal distension,vomiting.  T:37.5℃;HR:84bpm;BP:129/78mmHg | WBC:14.3×10⁹/L; CRP:98mg/L;  Lactate:0.8mmol/L;Procalcitonin:0.06ng/mL | Gastrointestinal decompression,  rehydration,PPIs,cephalosporin antibiotics,parenteral nutrition | Cefotaxime 1g q8h |
| Day 7  10:30 | Condition worsens | Abdominal pain worsened, fever  T:38.9℃;HR:118bpm;BP:94/52mmHg | WBC:18.9×10⁹/L; CRP:154mg/L;  Lactate:2.4mmol/L;Procalcitonin:2.1ng/mL | Oxygen administration, temperature reduction, and vasoactive drugs on standby | Cefotaxime 2g q8h |
| Day 7  11:00 | CT scan |  | CT:Gastrointestinal perforation, peritoneal effusion | Preoperative preparation | Cefotaxime 2g q8h |
| Day 7  12:00 | Laparoscopic surgery | T:38.2℃;HR:122bpm;BP:90/46mmHg |  | Kocher's operation,duodenal perforation repair,debridement of retroperitoneal abscess and drainage |  |
| Day 8  08:30 | Symptom relief | T:37.4℃;HR:92bpm;BP:121/74mmHg | WBC:14.4×10⁹/L; CRP:104mg/L;Lactate:1.0mmol/L | Oxygen administration,gastrointestinal decompression,rehydration,PPIs,cephalosporin antibiotics,parenteral nutrition | Cefotaxime 2g q8h |
| Day 10  12:30 | Bacterial culture results | T:37.6℃;HR:85bpm;BP:132/78mmHg. | Bacterial culture results: Escherichia coli (ESBL positive); Klebsiella pneumoniae | PPIs,antibiotics,parenteral nutrition | Meropenem 1g q8h |
| Day 12 | Clinical improvement | Abdominal pain significantly relieved;T:36.6℃;hemodynamically stable. |  | PPIs,antibiotics,enteral nutrition | Meropenem 1g q8h |
| Day 14 | CT scan | The drainage tube showed no fluid output. | WBC:7.6×10⁹/L; CRP:35mg/L;  Lactate:0.4mmol/L;procalcitonin:0.01ng/mL.  CT:The abscess cavity had shrunk to 2.1×1.5 cm, with no new effusion. | Removed the drainage tube; gradually resumed oral intake. | Cefixime 200 mg po bid |
| Day 16 | Discharged | No fever, no abdominal pain; tolerates full diet. |  | PPIs |  |
